# Evaluation of *in vivo *labelled dendritic cell migration in cancer patients

**DOI:** 10.1186/1479-5876-2-27

**Published:** 2004-07-30

**Authors:** Ruggero Ridolfi, Angela Riccobon, Riccardo Galassi, Gianluigi Giorgetti, Massimiliano Petrini, Laura Fiammenghi, Monica Stefanelli, Laura Ridolfi, Andrea Moretti, Giuseppe Migliori, Giuseppe Fiorentini

**Affiliations:** 1 Department of Medical Oncology, Morgagni-Pierantoni Hospital, Via Forlanini 34, 47100 Forlì, Italy; 2 Nuclear Medicine Unit, Morgagni-Pierantoni Hospital, Via Forlanini 34, 47100 Forlì, Italy; 3 Health Physics Unit, Morgagni-Pierantoni Hospital, Via Forlanini 34, 47100 Forlì, Italy; 4 Istituto Oncologico Romagnolo, Corso Mazzini 65, 47100 Forlì, Italy; 5 Blood Transfusion Unit, Morgagni-Pierantoni Hospital, Via Forlanini 34, 47100 Forlì, Italy

## Abstract

**Background:**

Dendritic Cell (DC) vaccination is a very promising therapeutic strategy in cancer patients. The immunizing ability of DC is critically influenced by their migration activity to lymphatic tissues, where they have the task of priming naïve T-cells. In the present study *in vivo *DC migration was investigated within the context of a clinical trial of antitumor vaccination. In particular, we compared the migration activity of mature Dendritic Cells (mDC) with that of immature Dendritic Cells (iDC) and also assessed intradermal versus subcutaneous administration.

**Methods:**

DC were labelled with ^99m^Tc-HMPAO or ^111^In-Oxine, and the presence of labelled DC in regional lymph nodes was evaluated at pre-set times up to a maximum of 72 h after inoculation. Determinations were carried out in 8 patients (7 melanoma and 1 renal cell carcinoma).

**Results:**

It was verified that intradermal administration resulted in about a threefold higher migration to lymph nodes than subcutaneous administration, while mDC showed, on average, a six-to eightfold higher migration than iDC. The first DC were detected in lymph nodes 20–60 min after inoculation and the maximum concentration was reached after 48–72 h.

**Conclusions:**

These data obtained *in vivo *provide preliminary basic information on DC with respect to their antitumor immunization activity. Further research is needed to optimize the therapeutic potential of vaccination with DC.

## Background

Dendritic Cell (DC) vaccination is one of the most promising tools of immunological therapy for cancer. Administration of DC, generated and loaded with tumor antigens *ex vivo*, can be used to circumvent tumor immunotolerance [1,2]. A large number of immature DC (iDC) can be produced by culturing peripheral blood monocytes with GM-CSF and IL-4 *in vitro. *These iDC possess functional characteristics typical of this maturation status, such as phagocytosis, macropinocytosis, receptor-mediated endocytosis and antigen processing [3,4]. After antigen uptake and processing, under inflammatory stimuli, iDC undergo functional changes that result in their maturation (mDC) [5]. Following the up-regulation of HLA class I and II and costimulatory molecules (CD80, CD86) and other specific markers such as CD83, DC-LAMP and CCR7, mDC migrate to the T-cell zone of lymphoid tissue, where they have an optimal stimulatory capacity [6,7]. The migration of DC to regional lymph nodes therefore represents one of the most important requirements for lymphocyte priming. Migration probably occurs through lymphatic pathways, but it is not known whether it is active or passive. Furthermore, factors such as PGE_2 _may considerably increase migration, inducing CCR7 expression on the surface of DC. Penetration may be limited to the peripheral zones of lymphoid tissue when the DC are still immature, or may reach the deeper T-cell zones, where a greater number of naïve T-cells are present, when DC are mature and activated.

Surface antigen CCR7, present on the cell membrane of DC, strongly influences migratory capacity through its interaction with transporter molecules, TREM-2, LTC4, LTD4, etc. [8-10]. The mDC that reach lymph nodes prime naïve T-cells for a limited time and then exhaust their active functions. This can be verified by measuring IL-12 production, which rapidly decreases, and by determining the presence of IL-10, previously absent. Special conditions such as the linkage with lymphocyte ligand CD40 may prolong the active phase of mDC [11-13].

Recent studies on cancer patients evaluating the efficacy of *in vitro*-generated vaccines have shown that mature, but not immature DC, induce an effective antitumor response [14-18]. Animal studies have provided direct evidence that subcutaneously injected DC preferentially migrate to draining lymph nodes to induce a measurable antitumor effect [18,19]. Similarly, the use of radiolabelled DC in humans demonstrates the ability of these cells to migrate to draining lymph nodes. It has also been observed that migration efficiency is linked to their maturation status or administration route (intravenous, subcutaneous, or intradermal) [20-23].

In the course of a vaccination trial using DC pulsed with autologous tumor lysate (ATL) in cancer patients, we evaluated the *in vivo *migration ability of DC by labelling them with ^99m^Tc-HMPAO or ^111^In-Oxine. In particular, migratory activity was assessed in iDC and mDC in terms of time required for migration to lymph nodes, duration of activity, and number of cells that migrated. Migratory capacity was further evaluated by comparing subcutaneous and intradermal administration.

## Materials and methods

### Patients

The case series consisted of a subset of the 19 patients enrolled onto a phase I/II vaccination trial for advanced melanoma and renal cell carcinoma in which the first 9 patients were treated with iDC and the remaining 10 received mDC, both pulsed with autologous tumor lysate and keyhole limpet hemocyanin (Biosyn, Fellbach, Germany). In the present study 8 patients were analyzed (7 melanoma, 1 renal carcinoma) for a total of 11 treatments. *In vivo *migration was assessed using a part of the DC obtained for one of the therapy cycles. Three of the 8 patients were evaluated twice.

Two melanoma patients were treated with iDC (one of whom twice), while 4 other patients with melanoma and 1 with renal cell carcinoma (treated twice) received mDC. The remaining melanoma patient was treated with iDC and subsequently with mDC.

The clinical trial was approved by the Italian Ministry of Health and by the Ethical Committee of Forlì Health and Social Services (Azienda USL – Forlì, Italy). All patients gave written informed consent.

### Tumor lysate

Tumor samples surgically removed from the patients were immediately placed in PBS. Adjacent non malignant tissue was removed by scalpel and tumor cells were dispersed to create a single-cell suspension. Cells were lysed by incubation in sterile distilled water. Lysis was monitored by light microscope. Larger particles were removed by centrifugation (10 min at 600 *g*) and the supernatant was passed through a 0.2-μm filter. Protein contents were determined and aliquots were stored at -80°C until use.

### Treatment

Patients were generally vaccinated intradermally with DC (4–6 inoculations at the base of the thigh, about 10 cm from the groin, in the absence of visible disease). From days 2–6, IL-2 (Chiron, Milan, Italy) was administered subcutaneously at a dose of 3 million IU/die. This procedure was repeated after two weeks and once a month until progression occurred.

### DC generation

DC were prepared from peripheral blood monocytes (PBMC) obtained by leukapheresis without previous mobilization. 5–9 liters of blood were processed in each collection. PBMC were purified on Ficoll-Paque. An aliquot of PBMC was utilized immediately for DC generation and the rest was frozen in bags for use at a later date (4–5 bags/1 collection).

PBMC were incubated in tissue culture flasks with CellGro DC Medium (Cell Genix, Freiburg, Germany) at 10 × 10^6 ^cells/ml for 2 h. The non-adherent cells were discarded and the adherent cells were incubated in CellGro DC Medium containing 1000 IU/ml rhIL-4 (Cell Genix) and 1000 IU/ml rhGM-CSF (Shering Plough, Milan, Italy) for 7 days to generate a DC-enriched cell population. On day 6 DC were pulsed with autologous tumor lysate (100 mg/ml) and with KLH (50 mg/ml) and incubated overnight. On day 7, they were defined as iDC. After eliminating the previous culture medium, pulsed iDC were cultured for a further 2 days with a cocktail of cytokines (TNFα, IL-1β, IL-6, Endogen, Pierce Biotechnology, Rockford, USA; PGE_2_, Cayman Chemical, Ann Arbor, MI, USA). On day 9 they were defined as mDC. iDC or mDc were removed, washed and suspended in sterile saline for therapeutic infusion into the patient.

### DC labelling and migration evaluation

Labelling of DC was performed according to the methods described for leucocyte radiolabelling [24-26]. A part of both iDC and mDC destined for vaccination (about 9.10^6^) were resuspended in platelet-poor autologous plasma (CFP1) and incubated for 15 min at room temperature with ^99m^Tc-HMPAO (20 mCi) (Nycomed Amersham plc, Little Chalfont, UK) ^111^In-Oxine (1 mCi) (Altana Pharma, Milan, Italy). After two washes to eliminate the unbound isotope, the cells were resuspended in a total volume of 1.5 ml of CFP1. Radiolabelling of the DC and of the culture supernatant was evaluated with a gamma counter, after which DC were inoculated intradermally into the patient near healthy lymph nodes and in the contralateral zone not used for therapeutic vaccination (3 inoculations at 10 cm from inguinal or axillary lymph nodes). The patient then underwent serial acquisitions with gamma-camera positioned at the site of inoculation, with a field of view that included all the lymphatic regions of interest. The first acquisition was performed with a dynamic study of 20 min, followed by 10-min static acquisitions carried out every 30 min for the first 4–6 h and from 18 to 28 h. Other static determinations were made at 36, 48 and 72 h. The maximum duration of observation of DC migratory activity, which depended on the half-life of the radioisotope used, was 72 h for ^111^In-Oxine and 36 h for ^99m^Tc-HMPAO.

The identification of lymph node stations involved in the migratory activity was initially visual, after which we carried out a semiquantitative evaluation of the percentage of DC that migrated to lymph nodes from the inoculation site and an assessment of the speed of DC migration, expressed by activity/time curves obtained through the compartmental mathematical model.

### Evaluation of labelling stability

DC obtained from the culture of frozen PBMC were divided into two parts: one was labelled with ^99m^Tc-HMPAO and the other was labelled with ^111^In-Oxine. The labelled cells were then suspended in CellGro DC Medium, divided into 4–5 culture flasks for each labelling molecule and incubated for 0 h, 4 h, 21 h, 24 h (^99m^Tc-HMPAO) and 0 h, 4 h, 21 h, 24 h, 48 h (^111^In-Oxine). The DC from one flask were removed and centrifuged. The supernatant containing the free molecule, and the pellet containing the labelled cells, were then measured with a gamma counter.

### Phenotype analysis

iDC and mDC phenotypes were determined by single or two-color fluorescence analysis. 3–5·10^5 ^cells were suspended in 100 μl of buffer (PBS, 2% FCS, 1% sodium azide) and incubated for 30 min at 4°C with 10 μl of appropriate fluorescein isothiocyanate or phycoerythrin-labelled monoclonal antibodies (mAbs). The cells were then washed twice and resuspended in 500 μl of assay buffer. The fluorescence was analyzed by a FACS Vantage flow cytometer (Becton Dickinson, Milan, Italy). mAbs specific for human CD1a, CD14, CD80, CD86, (Becton Dickinson) CD83 (Immunotech, Marseille, France) and CCR7 (BD Pharmingen, Milan, Italy) were used.

### Cytokine production

At each pre-set time the supernatant was collected and stored at -80°C until analysis was carried out using commercially available ELISA Kit (Biosource, Nivelles, Belgium) to measure the production of IL-12 + p40 (bioactive heterodimer of IL-12) and IL-10 by DC.

### Endocytosis evaluation

Single cell-based measurement of endocytosis was carried out as described (27). Dendritic cells were incubated for 30 min at 37°C with 0.5 mg/ml FITC-Dextran (40S DX-FITC Sigma, Milan, Italy). DX-FITC (average MW 42,000) was centrifuged before use to remove aggregates. As negative control, cells were incubated with DX-FITC at 4°C. The cells were washed with cold PBS containing 2% FCS and 2 nM sodium azide to exclude dead cells and were then analyzed on a FACS Vantage flow cytometer (Becton Dickinson) [27].

## Results

### Patient characteristics

All the patients (6 males, 2 females) had advanced disease and all but one had undergone previous treatment. Median age was 49 years (range 46–52 years). Three patients were HLA-A1, 3 were HLA-A3, 1 was HLA-A2 and 1 was HLA-A11 (Table 1). Two melanoma patients were treated with iDC, while 2 other patients with melanoma and 1 with renal cell carcinoma received mDC. The remaining 3 melanoma patients were treated with iDC and subsequently with mDC (Table 1). The 8 patients received a total of 73 therapeutic vaccination cycles (20 with DC obtained from fresh PBMC and 53 from frozen PBMC) and 11 labelled DC evaluations were carried out.

**Table 1:** Patient characteristics

**Patients**	**Sex/Age**	**Pathology**	**Site of Metastasis**	**Previous Treatment**	**i/mDC**	**HLA**
1	M/47	Mel	Liver, mediastinal lymph nodes	IFN	iDC	A_1_A_2_B_8_B_35_Bw_6_Cw_4_Cw_7_
2	M/52	Mel	Liver	BIOCT	iDC	A_3_A_28_B_35_B_53_Cw_4_
3	M/49	Mel	Liver, adrenal glands	No treatment	iDC + mDC	A_11_A_31_B_14_B_60_Bw_6_Cw_3_
4	M/42	Mel	Liver, mediastinal and axillary lymph nodes	BIOCT	iDC+ mDC	A_1_A_9_B_17_Bw_4_Bw_6_Cw_3_Cw_4_
5	F/49	Mel	Lung, lymph nodes, skin, peritoneum	BIOCT	iDC+ mDC	A_1_A_9_B_7_B_44_Bw_4_Bw_6_Cw_4_Cw_7_
6	M/50	Renal ca.	Skin, adrenal glands	BIOCT	mDC	A_2_A_3_B_7_B_51_Bw_4_Bw_6_Cw_1_Cw_7_
7	F/52	Mel	Lung, liver	HdIFN + CT	mDC	A_3_A_29_B_44_Bw_4_
8	M/46	Mel	Abdominal lymph nodes	IFN+BIOCT	mDC	A_3_A_28_B_21_B_35_Cw_4_

IFN, alpha interferon; BIOCT, biochemotherapy; CT, chemotherapy; HdIFN, high-dose adjuvant alpha interferon (ECOG 1684)

### DC characteristics

The characteristics of iDC and mDC used to evaluate migration activity were similar to those of the DC utilized by us for therapeutic vaccination and to results published in the literature. Data on the purity and vitality of DC, the presence of surface markers and DC functional features (endocytosis and production of IL-12 and IL-10) are reported in Table 2.

**Table 2:** Biological characteristics of dendritic cells used for vaccination

	**iDC median % (range)**	**mDC median % (range)**
**DC surface markers:**		
**CD 1a**	20 (4–58)	2 (0–8)
**CD 14**	3 (0–7)	2 (0–11)
**CD 80**	3 (1–23)	37 (27–87)
**CD 86**	30 (10–55)	81 (15–94)
**HLA-DR**	45 (17–82)	78 (56–88)
**CD 83**	2 (0–13)	55 (34–73)
**CCR7**	4 (2–5)	86 (48–92)
**Endocytosis **% of positive cells	70 (39–91)	15 (1–42)
**IL-12 production **pg/ml	49 (17–225)	> 1350
**IL-10 production **pg/ml	0	0
% purity *	74 (66–98)	59 (31–100)
% vitality **	75 (68–79)	82 (66–89)

* viable DC/viable DC + viable lymphocytes ** viable DC + viable lymphocytes/total cells

### DC labelling efficiency and stability

**Figure 1:**
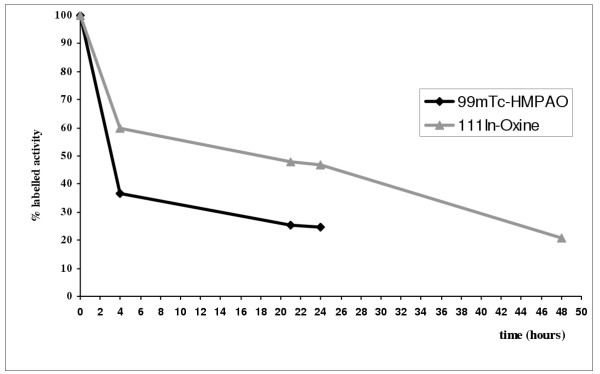
A sample of mature dendritic cells cultured *in vitro *for vaccination was divided into two parts, one labelled with ^99m^Tc-HMPAO and the other with ^111^In-Oxine. The DC were then suspended in DC medium and cultured *in vitro *for 24 h (^99m^Tc-HMPAO) and 48 h (^111^In-Oxine). At 0, 4, 21, 24, and 48 h, the activity of the supernatant containing the free molecule and of the pellet containing labelled cells was measured. After 24 h, a 75% and 50% loss of activity was observed for ^99m^Tc-HMPAO-and ^111^In-Oxine-labelled DC, respectively.

The *in vitro *stability of DC labelled with ^99m^Tc-HMPAO and ^111^In-Oxine was evaluated using DC cultured from frozen PBMC. ^99m^Tc-HMPAO-labelled DC showed a 75% loss of activity 4–24 h after the beginning of *in vitro *culture. ^111^In-Oxine-labelled DC showed a higher labelling stability (50%) that lasted for up to 24 h (Fig. 1). This accounts for the differences in lymph node uptake percentages observed in our migration studies. More accurate information on the linkage stability of ^111^In-Oxine-labelled DC over time would permit the opportune correction of the uptake percentage and would enable data to be compared with those obtained using indium.

### Administration routes

**Figure 2:**
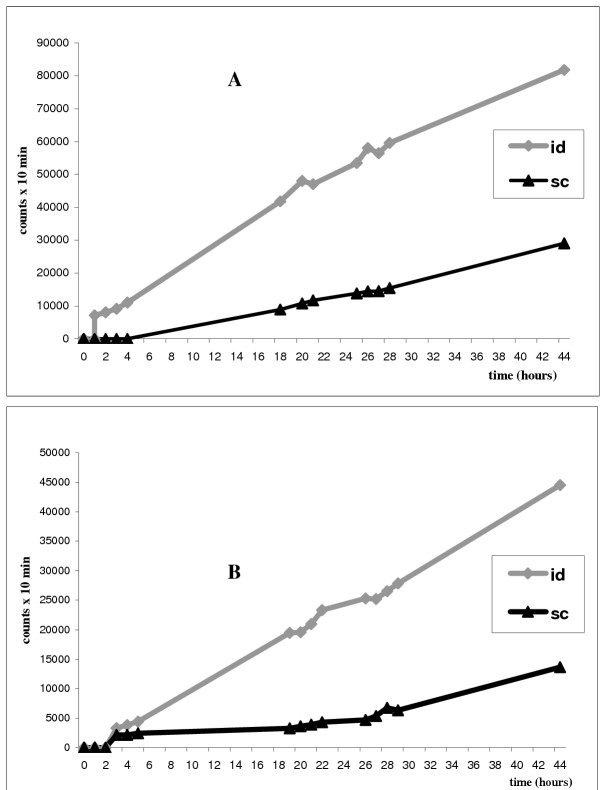
In patients no. 7 (A) and 8 (B), the same number of ^99m^Tc-HMPAO-labelled DC were administered simultaneously: subcutaneously (sc) in the left axilla and intradermally in the right axilla (id). The acquisition times with gamma camera are reported along the X-axis and the Y-axis shows the counts per 10 min. In both patients intradermal administration presents a greater concentration of labelled cells in lymph nodes than the subcutaneous route.

**Figure 3:**
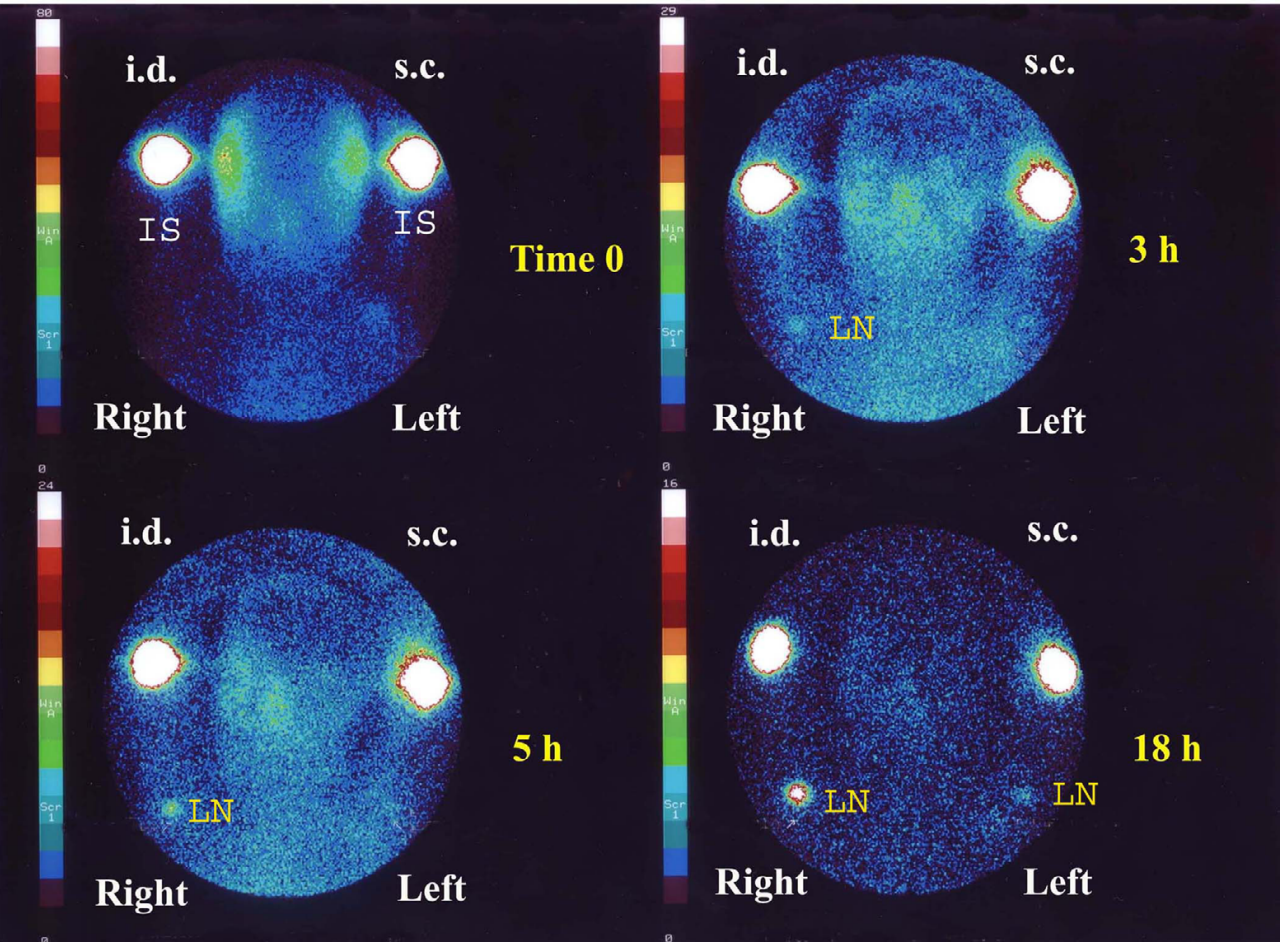
In patient no. 7 the same number of ^99m^Tc-HMPAO-labelled DC were administered simultaneously: subcutaneously (s.c.) in the left axilla and intradermally (i.d.) in the right axilla. The figure shows acquisition images with gamma camera at 0, 3, 5, and 18 h after inoculation. Greater migration capacity after intradermal administration is clearly visible. (IS, inoculation site: LN, lymph node).

The migration activity of mDC administered simultaneously by intradermal and subcutaneous injection in the arms of two patients (nos. 7 and 8) was evaluated by comparing radioactive uptake in axillary lymph nodes. The intradermal route showed a threefold higher migration than that observed for the subcutaneous route (Table 3). Evaluations were made at intervals from 0 to 44 h after inoculation. The sites of inoculation showed an exponential type washout that was virtually identical for both routes of administration (data not shown). The final migration percentage ratio (measured after 44 h) was fairly similar in both patients, but was obviously not statistically significant (Fig. 2A,2B,3).

**Table 3:** Different vaccine administration routes: intradermal vs. subcutaneous lymph node uptake

**Patients**	**mDC × 10^6^**	**Administration route**	**Isotope**	Max uptake (%) *
R.L. (no. 7)	4	Intradermal	**^99m^Tc-HMPAO**	0.95
G.D. (no. 8)	4	Intradermal	**^99m^Tc-HMPAO**	1.02
R.L. (no. 7)	4	Subcutaneous	**^99m^Tc-HMPAO**	0.30
G.D. (no. 8)	4	Subcutaneous	**^99m^Tc-HMPAO**	0.37

UPTAKE RATIO: intradermal/subcutaneous average: 3 *Maximum uptake = maximum lymph node activity/inoculation site activity at 0 h

### iDC/mDC migration

iDC and mDC migration was evaluated in all 8 patients (4 iDC and 7 mDC treatments) (Table 4). The data presented refer to 4 groups of patients treated with mature or immature cells labelled with ^111^In-Oxine or ^99m^Tc-HMPAO. A simple numerical analysis shows that the maximum uptake ratio between ^99m^Tc-HMPAO-labelled mDC and iDC varies from 2 to 35, with an average of 8.4. The same ratio for ^111^In-Oxine-labelled cells varies from 4 to 7, with an average of 6. ^99m^Tc-HMPAO labelling is influenced by the very low iDC uptake due to its greater binding instability and to the short half-life of the radioisotope, which does not permit the acquisition of reliable counts beyond 24–36 h.

**Table 4:** Comparison between iDC and mDC lymph node uptake

**Patients**	**DC i/m × 10^6^**		**Isotope**	Max uptake (%) *
G.C. (no. 1)	i	6	**^99m^Tc-HMPAO**	0.22
G.C. (no. 1)	i	6.9	**^99m^Tc-HMPAO**	0.05
G.L. (no. 2)	i	9	**^99m^Tc-HMPAO**	0.05
P.A.M. (no. 3)	i	6	**^111^In-Oxine**	0.42
T.N. (no. 5)	m	5.6	**^99m^Tc-HMPAO**	1.75
P.I.M. (no. 4)	m	12	**^99m^Tc-HMPAO**	0.53
PA.M. (no. 3)	m	7	**^111^In-Oxine**	3.14
S.G. (no. 6)	m	6.4	**^99m^Tc-HMPAO**	0.39
S.G. (no. 6)	m	6.7	**^111^In-Oxine**	1.88
R.L. (no. 7)	m	4	**^99m^Tc-HMPAO**	0.95
G.D. (no. 8)	m	4	**^99m^Tc-HMPAO**	1.02

**UPTAKE RATIO: mDC/iDC **^111^**In-Oxine **average: 6 (range 4–7) ^99m ^**Tc-HMPAO **average: 8.4 (range 2–35) *Maximum uptake = maximum lymph node activity/ inoculation site activity at 0 h

**Figure 4:**
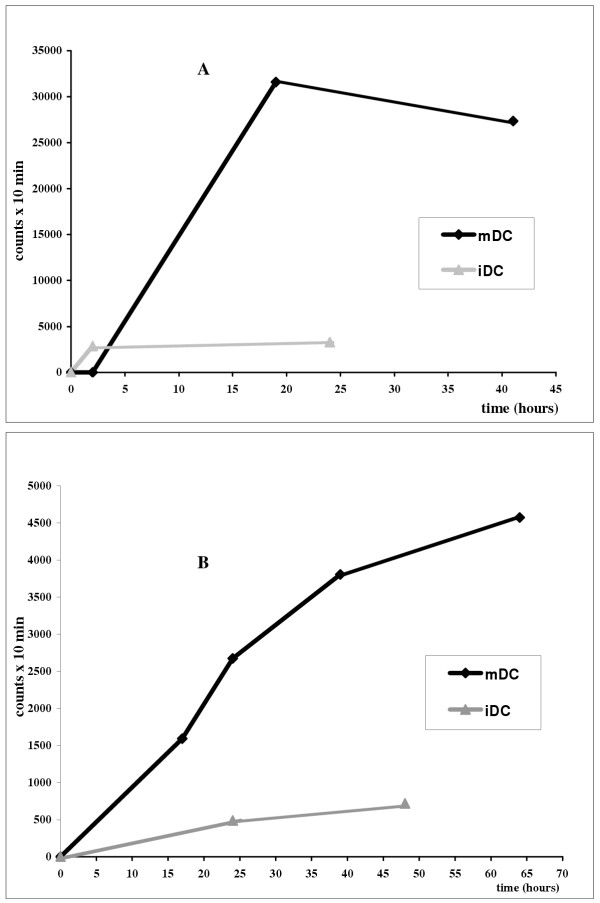
This figure shows the migration activity of mDC (patient no. 5) and iDC (patient no. 2) labelled with ^99m^Tc-HMPAO (A) and of mDC and iDC (patient no. 3) labelled with ^111^In-Oxine (B). The acquisition times with gamma camera are reported along the X-axis and the Y-axis shows the counts per 10 min. mDC migrating to regional lymph node are always higher than iDC. ^99m^Tc-HMPAO-labelled DC were detected in lymph nodes within 1 h of administration and the maximum concentration was reached within 60 min for iDC and between 18 and 20 h after inoculation for mDC.

**Figure 5:**
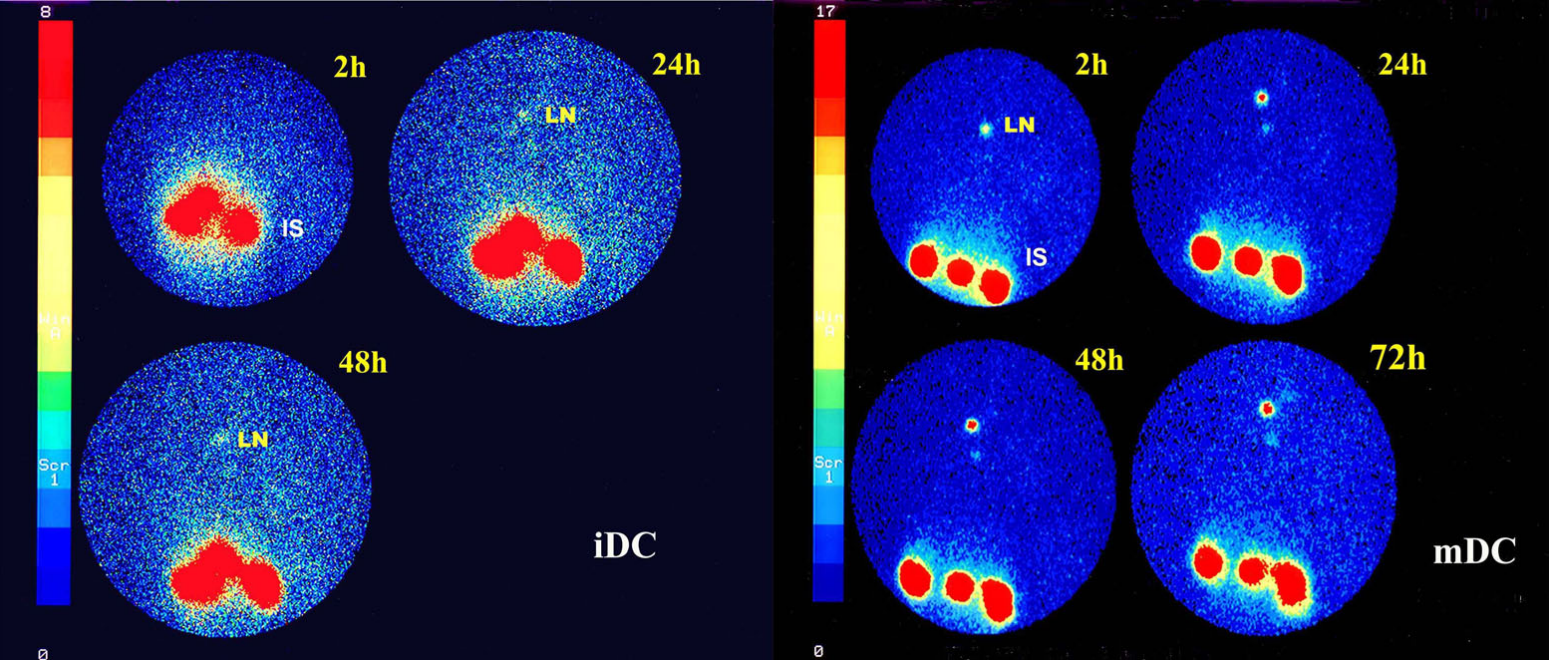
The figure shows static acquisition images with gamma camera 2, 24, and 48 h, and 2, 24, 48 and 72 h after inoculation with ^111^In-Oxine-labelled iDC and mDC, respectively, for patient no. 3. Greater migration activity of mDC is clearly visible. (IS, inoculation site: LN, lymph node).

A lymph node uptake can be observed in all patients within the first two hours of inoculation, reaching a maximum uptake after 12 h in 7 patients (Fig. 4A,4B,5). In the last 4 experiments in which a more accurate temporal analysis was performed, the uptake percentage continued to increase for the entire temporal range studied (24–30 h with ^99m^Tc-HMPAO and 48–60 h with ^111^In-Oxine). A curve fitting analysis also seemed to indicate a progressive increase in uptake after the first 60 h, but the number of patients evaluated is too low for any definitive conclusions to be drawn.

### Pure ^99m^Tc-HMPAO injection

After injection of ^99m^Tc-HMPAO alone in the same inoculation sites used for the study, no labelled hot spots were observed. This would seem to suggest that pure tracers move through lymphatic vessels without accumulating inside lymph nodes.

## Discussion

DC-based immunotherapy has undergone a remarkable transformation in its development from basic research to clinical application [10,28]. However, many issues remain to be clarified to improve functionality and therapeutic effects and to insure a powerful and wide-ranging antitumor response by T-cells. Two stages of fundamental importance in the therapeutic use of DC are the optimization of the maturation stimulus and the induction of an effective migration to regional lymph nodes to guarantee powerful and long-lasting priming of naïve T-cells [5,29]. Migration activity is therefore one of the functional characteristics of DC that warrants further investigation in an attempt to increase their potential [30].

Recent published data have shown that the choice of maturation stimulus may be crucial for therapeutic success. In particular, it has been seen that PGE_2 _is essential for activating DC chemotaxis through the expression of CCR7 on the cell surface [31,32]. This receptor permits migration through a concentration gradient of its ligands CCL19 and CCL21 inside lymphoid organs. The use of PGE_2 _may therefore prove to be important for increasing migration activity and DC efficacy. However, it has also been observed that PGE_2 _inhibits IL-12 production, resulting in a weaker *in vivo *activation of T-cells [33,34]. These contradictory research data obviously require validation by *in vivo *experimentation.

In the present study we aimed to clarify some issues concerning DC migration activity in a clinical vaccination trial utilizing radiolabelled (^99m^Tc-HMPAO and ^111^In-Oxine) iDC and mDC [20,35]. Technetium has a high labelling intensity and a short half-life, while indium, despite having a lower intensity, has a longer half-life, which enabled us to monitor the migration activity of labelled DC for up to 72 h.

The first step was to evaluate whether radioactive tracer would be scintigraphically visualized in lymph nodes. For this purpose technetium alone was injected intradermally and its progression followed. At the same time labelled DC were administered in the contralateral zone. It was seen that the tracer was not entrapped in lymph node stations and this confirmed that the radioactive molecule detected in contralateral regional lymph nodes was undoubtedly the expression of labelled DC that had migrated to that site.

One of the simplest analyses carried out was the evaluation of mDC migration activity using two different routes of administration: subcutaneous and intradermal. We chose two patients at random and compared migration activity simultaneously in both arms (right intradermal and left subcutaneous); it was observed that intradermal administration had a threefold higher migration to lymph nodes than the subcutaneous route. Although it remains to clarify the extent to which this migratory capacity is active or passive, it is clear that DC must be administered intradermally to obtain a higher migration.

A crucial phase of the study was the comparison between the migratory activity of iDC (used in the first part of the clinical trial) and that of mDC (used in the second half of our ongoing study). The result was once again unequivocal, showing a greater progressive concentration of mDC that was, on average, six-eightfold higher than that of iDC, in accordance with data reported by other authors [20,23] and in contrast to results published by Blocklet [17], who probably used iDC. The phenotype obtained in our study bears witness to the fact that mDC exhibit a much higher CCR7 surface expression than iDC (86% vs. 4%) (Table 2). Furthermore, mDC have an extremely high IL-12 production, which confirms a marked stimulatory activity.

Notwithstanding the results obtained from the present study, many issues remain to be clarified. It has yet to be determined whether the increased activity detected in lymph nodes corresponds to an effectively greater migratory capacity or whether it is the result of a more effective adhesion capacity between surface molecules. Both hypotheses could even prove to be correct. We also do not know how long DC remain in lymph nodes. The increase in activity in lymph nodes is high in the first few determinations but tends to diminish or stabilize after around 36 h. It remains to be seen whether this presumed stabilization is the result of a sort of saturation or whether it can be attributed to the attainment of a dynamic equilibrium. The former hypothesis would indicate the need for an optimization of the number of DC to administer, as only a limited number would be functionally active. The latter would highlight the importance of the timing of administration and perhaps also the degree of DC maturation. To further investigate this, we plan to administer ^111^In-Oxine-and ^99m^Tc-HMPAO-labelled DC in succession and in the same site, to follow their migratory course.

Finally, we aim to assess the migratory capacity of *in vitro *transiently stimulated DC (semimature DC). The therapeutic use of this type of DC, which have already begun the process of maturation and may be capable of reaching lymph nodes before their functional exhaustion, could increase the duration of their activation and stimulation. If these semimature DC prove to be equipped with a good migratory capacity, further improvement in the therapeutic use of DC may be possible.

## Conclusions

The migration activity of DC to regional lymph nodes is one of the many critical factors that influence the therapeutic result of antitumor vaccination. In the present study we used radioisotope-labelled DC and demonstrated that a better migration activity is obtained using intradermal than subcutaneous administration and that mDC show, on average, a six-to eightfold higher migration than iDC. Numerous other issues on DC functionality have yet to be clarified before antitumor therapeutic efficacy can be improved. The next important step will be to closely monitor the quantity and quality of responses observed in T-cells, and it is hoped that a consensus will be reached on standardized criteria for the definition and validation of clinical results obtained.

## Abbreviations

DC, dendritic cell; iDC, immature dendritic cell; mDC, mature dendritic cell; ATL, autologous tumor lysate; PBMC, peripheral blood monocytes.

## Authors's contributions

RR and LR participated in the design of the study and were responsible for the clinical side of the study. AR, MP, LF and MS participated in the design of the study and were responsible for the biological part of the study. GM performed the apheresis collections. RG, AM and GF carried out DC labelling and migration evaluation. GG performed the mathematical and statistical analysis. All authors read and approved the final manuscript.

## Competing interests

None declared.
